# Empirical antibiotic therapy in acute orthopaedic infections: differences in antimicrobial susceptibility across anatomical sites

**DOI:** 10.5194/jbji-11-229-2026

**Published:** 2026-04-15

**Authors:** Yoni Lodewijk-van den Brink, Jon H. M. Goosen, Denise S. C. Telgt, Marrigje Nabuurs-Franssen, Karin C. M. Veerman

**Affiliations:** 1 Department of Orthopaedics, Sint Maartenskliniek, Nijmegen, the Netherlands; 2 Department of Medical Microbiology, Canisius Wilhelmina Hospital, Nijmegen, the Netherlands

## Abstract

**Background:** The selection of empirical antibiotic therapy for early postoperative orthopaedic infections is challenging because pathogen distribution, antimicrobial resistance, and microbiology vary locally and by anatomical site. Dutch national guidelines from the Dutch Working Party on Antibiotic Policy (SWAB) recommend vancomycin-based combination therapy for prosthetic joint infection (PJI), but the adequacy of these regimens for the broader spectrum of early postoperative orthopaedic infections has not been systematically evaluated. **Methods:** We conducted a retrospective, single-centre cohort study including early postoperative orthopaedic infections ( ≤ 90 d) treated between January 2022 and January 2025 with complete microbiological- and antimicrobial-susceptibility data. Microbiological isolates, susceptibility profiles, anatomical location, and the type of index procedure were analysed. Empirical coverage was assessed for cefazolin, vancomycin–ceftriaxone (SWAB-recommended), and vancomycin–ciprofloxacin, both overall and stratified by anatomical site. **Results:** A total of 304 infections were included. Gram-positive organisms predominated, including *Staphylococcus aureus* (one MRSA isolate) and coagulase-negative staphylococci, alongside Gram-negative bacteria, mainly *Enterobacter* spp. Despite high cefazolin susceptibility among *S. aureus*, cefazolin monotherapy covered only 49 % of infections due to resistance in *S. epidermidis* and Gram-negative pathogens. Vancomycin–ceftriaxone provided 89.8 % coverage and increased to approximately 93.4 % when foot and ankle infections were excluded; this subgroup showed frequent ceftriaxone-resistant *Enterobacter* spp. Vancomycin–ciprofloxacin achieved the highest coverage (97.7 %). **Conclusion:** Cefazolin monotherapy is insufficient as an empirical treatment for early postoperative orthopaedic infections at our centre. Vancomycin–ceftriaxone offers high coverage for most anatomical sites and aligns with SWAB recommendations. For foot and ankle infections, vancomycin–ciprofloxacin offers superior coverage.

## 1 Introduction

Implant-related infections are serious complications in orthopaedic surgery. The reported incidence of periprosthetic joint infections (PJIs) after total joint arthroplasty is approximately 2 % (Goud et al., 2023; Tande and Patel, 2014), while infection rates after fracture fixation vary between 1 % and 30 %, depending on the fracture type, location, and patient factors (Metsemakers et al., 2018; Trampuz and Zimmerli, 2006). Both conditions are notoriously difficult to treat and often require complex surgical and antimicrobial strategies provided by a multidisciplinary team.

The treatment of PJI is complex and depends on multiple factors, including timing (early vs. late), clinical presentation (acute vs. chronic), patient comorbidities, implant stability, and pathogen type. Common strategies include debridement; antibiotics and implant retention (DAIR); one- or two-stage revision arthroplasty; and, in rare cases, implant removal without reimplantation, chronic suppressive antibiotic therapy, or amputation (van Veghel et al., 2024; Aftab et al., 2025). Targeted antimicrobial therapy requires accurate microbiological identification to optimize antibiotic selection and clinical outcomes (Parvizi et al., 2011).

To reduce treatment variation and improve quality of care, the Dutch national guideline on antimicrobial treatment of prosthetic joint infection, issued by the Dutch Working Party on Antibiotic Policy (SWAB), was recently published (SWAB, 2024). The recommendations were informed by international evidence and tailored to surgical strategies and national epidemiologic data on antimicrobial resistance (Tande and Patel, 2014; Zimmerli et al., 2004; RIVM, 2024).

The Dutch guideline focuses exclusively on PJI, whereas orthopaedic practice also encompasses non-prosthesis-related infections such as osteomyelitis, post-traumatic infections, and infections after internal fixation (Lew and Waldvogel, 2004; Metsemakers et al., 2018). These conditions can result in comparable morbidity, prolonged treatment, and significant healthcare costs.

At the Sint Maartenskliniek, a tertiary referral centre for orthopaedic infections and one of the highest-volume centres for revision surgery and DAIR procedures in Europe, the newly introduced national recommendations for empirical therapy of acute PJI (vancomycin combined with ceftriaxone) differed markedly from the regimen previously in use (cefazolin). Because these guidelines were expected to substantially impact clinical practice and antimicrobial decision-making, we conducted a retrospective analysis of early postoperative orthopaedic infections, both prosthetic and non-prosthetic, treated at the Sint Maartenskliniek over the last 3 years.

The aim of this study is to identify local epidemiology and antimicrobial-susceptibility profiles to evaluate the alignment of the new Dutch national guideline with current clinical practice and to determine where additional or extended guidance may be required.

## 2 Methods

This retrospective, observational, single-centre study was conducted at the Sint Maartenskliniek (SMK), a specialized orthopaedic hospital in Nijmegen, the Netherlands. All patients treated for an early postoperative ( ≤ 90 d) infection following an orthopaedic procedure between January 2022 and January 2025, with complete microbiological- and antimicrobial-susceptibility data, were included in the analysis. In total, 304 early postoperative orthopaedic infections were analysed. Perioperative antibiotic prophylaxis at our centre consisted of cefazolin in accordance with national guidelines. In selected cases (e.g. β -lactam allergy), alternative agents were used according to local protocols during the study period. In addition, empirical antibiotic treatment for suspected early postoperative infections in clinical practice consisted of cefazolin monotherapy throughout the study period. The evaluation of alternative empirical regimens (e.g. vancomycin-based combinations) represents a theoretical analysis based on the observed microbiological-susceptibility data, and these regimens were not administered to patients in this cohort.

The cohort included both implant-related infections (prosthetic joint infections and infections after osteosynthesis) and infections following procedures without implanted material (e.g. arthroscopic or soft-tissue procedures). In addition, early hematogenous prosthetic joint infections treated with a DAIR procedure were included. Infections without a recent orthopaedic procedure, such as primary vertebral osteomyelitis, diabetic foot infections, native joint septic arthritis, and hematogenous osteomyelitis without implants, were not included.

Infections were categorized by anatomical location (hip, knee, upper extremity, foot/ankle, spine) and by type of index procedure (primary versus revision arthroplasty, osteosynthesis procedures). In addition, infections were categorized according to the presence of implanted material at the time of infection. Periprosthetic joint infections (PJIs) involved prosthetic implants. OSM + referred to infections after osteosynthesis procedures with retained implant material. OSM - referred to infections following orthopaedic procedures without implanted material at the time of infection, including procedures such as clavicle resection, lumbar laminectomy, and other soft-tissue or bone procedures without hardware.

Classification was based on implant status at the time of infection and not on the surgical treatment strategy.

### 2.1 Data collection and definitions

Data were retrospectively extracted from the electronic patient records, including patient demographics and comorbidities, procedure characteristics, clinical presentation, microbiological results, and clinical outcomes. An infection was diagnosed when ≥ 2 intraoperative tissue samples obtained during a debridement procedure yielded identical microorganisms or when a single sample yielded a highly virulent organism (e.g. *Staphylococcus aureus* or a Gram-negative bacillus). Orthopaedic tissue cultures were inoculated on both solid and broth media and incubated for 10–14 d; broth cultures were sub-cultured when turbidity developed. Microorganisms were identified using matrix-assisted laser desorption/ionization time-of-flight mass spectrometry (MALDI-TOF MS). Antimicrobial-susceptibility testing was interpreted according to EUCAST clinical breakpoints. Complete antimicrobial-susceptibility data were available for 304 of the 317 infections (96 %). Cases with incomplete resistance data ( n=13 ) were excluded from susceptibility and empirical coverage analyses.

After a DAIR procedure for suspected acute prosthetic joint infection, empirical antibiotic treatment in daily clinical practice consisted of cefazolin monotherapy throughout the entire study period, without changes in empirical treatment strategy. Based on antimicrobial-susceptibility data observed during the study period, hypothetical alternative regimens were evaluated for their potential adequacy if they had been administered instead of cefazolin. No patients in this cohort received these alternative empirical regimens. At the time of the study, updated national (SWAB) guidelines recommended broader empirical coverage; however, these recommendations had not yet been implemented at our centre during the study period.

Antimicrobial mismatch was defined as at least one causative microorganism being resistant to the empirical regimen. To assess potential mismatch rates with alternative empirical strategies, vancomycin plus ceftriaxone and vancomycin plus ciprofloxacin were evaluated retrospectively based on observed antimicrobial-susceptibility profiles.

## 3 Results

A total of 304 early postoperative orthopaedic infections with complete antimicrobial-susceptibility data were included in the analysis. Baseline patient characteristics are summarized in Table 1. The distribution of key comorbidities across anatomical infection sites was broadly comparable, with no major differences observed (Table 2). Of these infections, 152 (50 %) were periprosthetic joint infections (PJIs), 55 (18 %) were infections after osteosynthesis without retained implant material (OSM - ), and 97 (32 %) were infections after osteosynthesis with retained implant material (OSM +) .

**Table 1:** Baseline patient and infection characteristics ( n=304 ).

Category	Characteristic	Value
Demographics	Age, year – median (IQR)	62 (57–73)
	BMI, kg m^−2^ – median (IQR)	30 (25–34)
Comorbidities	Diabetes mellitus, n (%)	45 (15 %)
	Rheumatic disease, n (%)	61 (20 %)
	Immunosuppressive therapy, n (%)	31 (10 %)
	Active smoker, n (%)	41 (13 %)
Infection type	Periprosthetic joint infection (PJI), n (%)	152 (50 %)
	OSM $-{}^{a}$ , n (%)	55 (18 %)
	OSM $+{}^{b}$ , n (%)	97 (32 %)

^a^ OSM - Infection after osteosynthesis without retained implant material. ^b^ OSM + Infection after osteosynthesis with retained implant material.

**Table 2:** Distribution of comorbidities across anatomical infection sites.

Anatomical site	Diabetes, n	Rheumatic disease,	Immunosuppressive
	(%)	n (%)	therapy, n (%)
Hip ( n = 73)	8 (11.0 %)	12 (16.4 %)	11 (15.1 %)
Knee ( n = 49)	5 (10.2 %)	6 (12.2 %)	3 (6.1 %)
Spine ( n = 53)	11 (20.8 %)	10 (18.9 %)	1 (1.9 %)
Foot/ankle ( n = 63)	9 (14.3 %)	14 (22.2 %)	7 (11.1 %)
Upper extremity ( n = 66)	12 (18.2 %)	19 (28.8 %)	9 (13.6 %)

### 3.1 Microbiology

Most infections were caused by Gram-positive bacteria ( n=248 , 82 %), predominantly *Staphylococcus aureus* ( n=120 , 39 %) and coagulase-negative staphylococci (CoNS; n=83 , 27 %). Among the CoNS isolates, *S. epidermidis* was most frequent ( n=49 , 59 % of CoNS), followed by *S. capitis*, *S. hominis*, and *S. lugdunensis*. Other common Gram-positive organisms included *Cutibacterium acnes* ( n = 43, 14 %), *Enterococcus* spp. ( n=21 , 7 %), and *Streptococcus* spp. ( n = 29, 10 %).

Gram-negative bacteria were isolated in 76 cases (25 %), mainly *Enterobacter* spp. ( n = 46, 61 % of Gram-negative isolates), *Pseudomonas* spp. ( n = 22, 29 %), and *Escherichia coli* ( n = 8, 11 %). Additional isolates included *Klebsiella* spp. and several less frequently encountered species. Anaerobic or rare organisms, such as *Finegoldia magna* and *Corynebacterium* spp., were identified in six cases (2 %). No fungal or mycobacterial pathogens were identified in this cohort. Polymicrobial infections occurred in 99 cases (33 %). Because polymicrobial infections could contain both Gram-positive and Gram-negative pathogens, these categories were not mutually exclusive.

**Figure 1:**
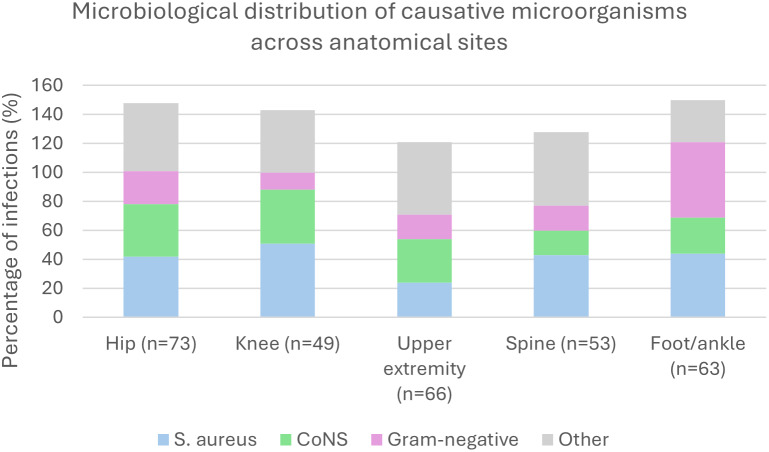
Distribution of causative microorganisms across anatomical infection sites. Pathogen groups are not mutually exclusive; therefore, percentages may exceed 100 % due to the presence of polymicrobial infections.

The distribution of causative microorganisms across anatomical sites is presented in Fig. 1. Gram-positive pathogens predominated in hip, knee, upper-extremity, and spine infections, mainly *Staphylococcus aureus* and coagulase-negative staphylococci. In contrast, foot and ankle infections showed a higher proportion of Gram-negative organisms, particularly *Enterobacter* spp., compared with other anatomical sites. Upper-extremity infections were relatively more often associated with other Gram-positive organisms, including *Cutibacterium* spp.

### 3.2 Antimicrobial susceptibility

A total of 304 infections had complete antimicrobial-susceptibility data and were included in the susceptibility and empirical coverage analyses. Among these infections, 149 (49 %) of the causative microorganisms were susceptible to cefazolin as empirical therapy. Cefazolin resistance occurred in 55 of 185 *Staphylococcus* isolates (30 %), nearly all of which were *S. epidermidis* (49 out of 55; 89 %). Only one methicillin-resistant *Staphylococcus aureus* isolate was identified.

If the combination of vancomycin and ceftriaxone had been used as empirical therapy, 273 of 304 infections (90 %) would have been susceptible. Gram-negative organisms were particularly common in foot and ankle infections, consistent with the distribution shown in Fig. 1, where ceftriaxone-resistant *Enterobacter* spp. were frequently observed. Excluding foot and ankle infections increased the empirical coverage of vancomycin–ceftriaxone to 225 out of 241 infections (93.4 %). The alternative combination of vancomycin and ciprofloxacin provided the highest empirical coverage, with 297 out of 304 infections (98 %).

**Table 3:** Empirical antimicrobial coverage by regimen and infection type.

Empirical regimen	PJI ( n = 152)	OSM - ( n = 55)	OSM + ( n = 97)
Cefazolin	71 (46.7 %)	40 (72.7 %)	38 (39.2 %)
Vancomycin–ceftriaxone	141 (92.8 %)	55 (100 %)	77 (79.4 %)
Vancomycin–ciprofloxacin	146 (97.3 %)	55 (100 %)	96 (98.9 %)

PJI denotes periprosthetic joint infection, OSM - denotes osteosynthesis without retained implant material, and OSM + denotes osteosynthesis with retained implant material. Values are presented as the number of infections with adequate empirical coverage ( n ) and the percentage of the total number of infections per group.

### 3.3 Antimicrobial susceptibility by anatomical group

Empirical antimicrobial coverage varied by anatomical location. In hip infections ( n = 73), cefazolin coverage was 43 %, vancomycin–ceftriaxone coverage was 95 %, and vancomycin–ciprofloxacin coverage was 96 %. Knee infections ( n = 49) showed susceptibility rates of 53 %, 92 %, and 94 %, respectively. In spinal infections ( n = 53), susceptibility rates were 53 % for cefazolin, 96 % for vancomycin–ceftriaxone, and 100 % for vancomycin–ciprofloxacin.

Foot and ankle infections ( n = 63) showed lower cefazolin coverage (40 %). Coverage increased to 76 % for vancomycin–ceftriaxone and 98 % for vancomycin–ciprofloxacin. Upper-extremity infections ( n = 66) showed coverage rates of 59 %, 91 %, and 100 %, respectively.

When categorized by procedure type, cefazolin coverage ranged from 39 % to 53 % after osteosynthesis procedures and reached 49 % in PJI. Coverage with vancomycin–ceftriaxone remained high across all subgroups (76 %–96 %), with particularly favourable rates in hip and knee infections (approximately 95 %). In foot and ankle infections, coverage increased substantially with vancomycin-based regimens, approaching near-complete coverage with vancomycin–ciprofloxacin (98 %). These findings are summarized in Table 4.

**Table 4:** Empirical antimicrobial coverage by anatomical group ( n = 304).

Anatomical site ( n )	Cefazolin	Vancomycin–ceftriaxone	Vancomycin–ciprofloxacin
	covered, n (%)	covered, n (%)	covered, n (%)
Total cohort ( n = 304)	149 (49.0 %)	273 (89.8 %)	297 (97.7 %)
Hip ( n = 73)	31 (42.5 %)	69 (94.5 %)	70 (95.9 %)
Knee ( N = 49)	26 (53.1 %)	45 (91.8 %)	46 (93.9 %)
Spine ( n = 53)	28 (52.8 %)	51 (96.2 %)	53 (100.0 %)
Foot/ankle ( n = 63)	25 (39.7 %)	48 (76.2 %)	62 (98.4 %)
Upper extremity ( n = 66)	39 (59.1 %)	59 (90.8 %)	65 (100.0 %)

## 4 Discussion

Our study shows that vancomycin–ciprofloxacin achieved the highest empirical antimicrobial coverage across all anatomical sites. However, vancomycin–ceftriaxone provides a more balanced empirical regimen for most anatomical sites when antimicrobial stewardship principles are taken into account. The lower empirical coverage of vancomycin–ceftriaxone in foot and ankle infections can be explained by the high prevalence of ceftriaxone-resistant *Enterobacter* spp. in this subgroup. For these infections, vancomycin–ciprofloxacin achieved near-complete coverage and may therefore be considered to be the preferred empirical regimen.

Given the ecological impact and rapid resistance selection associated with fluoroquinolones, particularly in high-inoculum infections, their use should be restricted to anatomical regions where broader Gram-negative coverage is clinically justified. In settings with higher rates of fluoroquinolone resistance among Gram-negative organisms, the empirical coverage of vancomycin–ciprofloxacin may be substantially lower than observed in our cohort.

Alternative broader-spectrum regimens, such as vancomycin–cefepime or vancomycin–carbapenem combinations, may further increase empirical coverage, particularly against *Pseudomonas* spp. and AmpC-producing Enterobacterales. However, their routine use should be carefully considered in light of ecological disadvantages. These regimens were not included in our empirical coverage analysis as they were not part of routine empirical therapy at our centre during the study period, and susceptibility data for these combinations were not consistently available. In line with antimicrobial stewardship principles, empirical therapy should be as narrow and as short in duration as possible and should be guided by local microbiological epidemiology.

The predominance of *Staphylococcus aureus* and coagulase-negative staphylococci in early postoperative or implant-related infections is consistent with several international reports (Trampuz and Zimmerli, 2006; Li et al., 2018). CoNS, particularly *S. epidermidis*, are known to exhibit β -lactam resistance (Arciola et al., 2018), supporting the rationale for glycopeptide-based empirical regimens. Our findings are also in line with Dutch data: Veerman et al. reported frequent antimicrobial mismatches in early PJI, largely driven by resistant staphylococci (Veerman et al., 2022), while Jacobs et al. highlighted the substantial clinical burden of infection as a major cause of failure in septic revisions (Jacobs et al., 2025). Together, these parallels suggest that the microbiological landscape of Dutch orthopaedic infections is comparable to international patterns, although reported resistance rates appear to be lower in Dutch cohorts.

Compared with a published Dutch study by Scholten et al., which evaluated empirical therapy for early PJI in hips and knees, our study included a broader range of postoperative infections, PJIs, osteosynthesis-related infections, and infections without implants across multiple anatomical sites (Scholten et al., 2023). Consequently, our dataset provides a more comprehensive basis for informing empirical treatment strategies across a broader orthopaedic population.

The limited empirical coverage of cefazolin in our cohort was attributable to both β -lactam-resistant Gram-positive cocci and the presence of Gram-negative bacteria. Similar observations have been described in other studies of orthopaedic infections, where early postoperative infections frequently involve mixed or Gram-negative flora (Metsemakers et al., 2018; Tande and Patel, 2014). In our study, *Enterobacter* spp. accounted for more than half of all Gram-negative isolates and are frequently resistant to ceftriaxone (EUCAST, 2024; Li et al., 2018). Ceftriaxone may be suboptimal for organisms with inducible AmpC β -lactamases, such as *Enterobacter cloacae* complex and *Klebsiella aerogenes*, for which broader-spectrum β -lactams (e.g. cefepime or piperacillin–tazobactam) may provide more reliable coverage. This finding provides a clear explanation for the lower empirical coverage of vancomycin–ceftriaxone in foot and ankle infections. When this anatomical subgroup was excluded, coverage increased from approximately 90 % to 93.4 %, indicating that the combination performs well across all other anatomical sites. These findings align with the overall superior empirical coverage observed for vancomycin–ciprofloxacin, although its broader ecological impact warrants caution in routine use.

The anatomical differences in microbiological profiles observed in our cohort align with previously reported location-specific patterns. Metsemakers et al. and Lipsky et al. described a higher prevalence of Gram-negative organisms in foot and ankle infections, likely related to local soft-tissue characteristics, colonization pressure, and vascular supply (Metsemakers et al., 2018; Lipsky et al., 2012). Although differences in patient characteristics may partly contribute to these findings, the distribution of key comorbidities, including diabetes mellitus, was comparable across anatomical sites in our cohort. In addition, perioperative antibiotic prophylaxis was standardized across procedures, making it unlikely that differences in prophylactic strategies explain the observed variation in microbiological profiles. Our findings support this observation and underscore the importance of tailoring empirical therapy to anatomical sites and local resistance patterns rather than applying a uniform regimen across all orthopaedic infections. Therefore, other factors, such as local tissue characteristics, colonization pressure, and anatomical differences, are more likely to explain the higher proportion of Gram-negative organisms.

This study has several limitations. Its single-centre design may limit the generalizability of the findings as local microbiological epidemiology and resistance patterns can vary between institutions. In addition, the low prevalence of methicillin-resistant *Staphylococcus aureus* (MRSA) in our cohort may limit the generalizability of our findings to settings with higher MRSA rates, such as southern Europe or other regions with different resistance epidemiology.

Although treatment strategies differ between implant-related and non-implant-related infections, our analysis focused on microbiological susceptibility and empirical antimicrobial coverage, which are relevant across these groups. Furthermore, the retrospective design introduces the risk of selection bias and limits the availability of detailed clinical data. In addition, the empirical antibiotic regimens evaluated in this study were assessed retrospectively based on microbiological-susceptibility data and therefore represent theoretical estimates of empirical coverage. Finally, clinical outcomes were not included in this analysis, and, therefore, no conclusions can be drawn regarding the relationship between empirical antimicrobial coverage and treatment success. However, this study was conducted at a high-volume tertiary referral centre with extensive experience in the management of early postoperative orthopaedic infections and DAIR procedures, which strengthens the clinical relevance and reliability of the findings.

Our findings also highlight that microbiological epidemiology is context-dependent and that local pathogen distribution and resistance patterns should be considered when selecting empirical antibiotic strategies.

Although the importance of timely and adequate antimicrobial therapy is well established in severe infections such as sepsis, where inadequate empirical therapy has been associated with worse outcomes (Evans et al., 2021; Paul et al., 2010), evidence specifically addressing the impact of short-term empirical mismatch in postoperative orthopaedic infections remains limited. In orthopaedic infections, treatment success is primarily determined by adequate surgical management, including timely debridement, in combination with targeted antimicrobial therapy once culture results become available (Zimmerli et al., 2004; Tande and Patel, 2014). The independent impact of a brief period of inadequate empirical coverage on outcomes following DAIR procedures is therefore still uncertain. Future studies incorporating clinical endpoints are needed to clarify whether improving empirical antimicrobial coverage translates into better patient outcomes. Accordingly, our study therefore focuses on microbiological coverage as a first step in optimizing empirical treatment strategies.

The strengths of this study include the large sample size, comprehensive microbiological data across multiple anatomical sites, and inclusion of different procedure types within a high-volume orthopaedic centre. Limitations include its retrospective design and the potential underrepresentation of culture-negative infections. Despite these limitations, the presented susceptibility data provide clinically relevant insights that can help refine empirical antibiotic policies for early postoperative orthopaedic infections.

## 5 Conclusion

When anatomical location is not taken into account, our findings are consistent with the new Dutch national guideline and show that vancomycin–ceftriaxone provides adequate empirical coverage for most early postoperative orthopaedic infections. However, when stratified by anatomical location, our results support reconsideration of empirical treatment strategies, particularly for foot and ankle infections where ceftriaxone-resistant *Enterobacter *spp. are prevalent. In this subgroup, vancomycin–ciprofloxacin offers superior empirical coverage and may therefore be considered to be the preferred empirical regimen. These findings highlight the importance of tailoring empirical antibiotic therapy to anatomical site and local microbiological epidemiology. Future studies should evaluate the clinical impact of these strategies.

## Data Availability

The data that support the findings of this study are not publicly available due to privacy and ethical restrictions but are available from the corresponding author upon reasonable request.
